# Identification of Candidate Genes Associated with the Pileus-Deficient Phenotype in *Lentinula edodes* Through Comparative Genomic and Transcriptomic Analyses

**DOI:** 10.3390/jof12050328

**Published:** 2026-05-01

**Authors:** Bo-Min Seo, Che-Hwon Park, Sung-Chul Lee, Rae-Won Kang, Young-Jin Park

**Affiliations:** 1 Department of Medicinal Biosciences, College of Biomedicinal and Health Science, Konkuk University, Chungju 27478, Republic of Korea; sbomin24@kku.ac.kr (B.-M.S.); chehwon9798@kku.ac.kr (C.-H.P.); vwm963@kku.ac.kr (S.-C.L.); fodnjs72617@kku.ac.kr (R.-W.K.); 2 Natural Product Bio-Convergence Research Center, Konkuk University, Chungju 27478, Republic of Korea

**Keywords:** *Lentinula edodes*, fruiting body development, transcriptome analysis, major facilitator superfamily, presence–absence variation

## Abstract

This study aimed to elucidate the molecular mechanisms underlying phenotypic divergence between two *Lentinula edodes* strains, Le_L and Le_S, which exhibit distinct fruiting body morphologies. While phenotypic variation among mushroom strains has been widely observed, the relative contributions of transcriptional regulation and structural genomic variation to these differences remain poorly understood. Comparative transcriptome analysis identified 8541 differentially expressed genes (DEGs), revealing clear functional divergence between the two strains. Genes upregulated in Le_S were predominantly enriched in ribosomal components and translation-related processes, indicating enhanced protein synthesis activity. In contrast, Le_L-upregulated genes were associated with transporters, transcription factors, and diverse metabolic pathways, suggesting broader regulatory and physiological functions. Protein–protein interaction network analysis further highlighted distinct regulatory architectures, with ribosomal proteins forming highly interconnected hub gene modules in Le_S, whereas Le_L hub genes were functionally diverse and included multiple members of the Major Facilitator Superfamily (MFS). Ortholog analysis across 33 *L. edodes* strains demonstrated that most hub genes were conserved, indicating their roles as core genetic components. Despite widespread genome-wide variation, including 7931 SNPs and 1149 INDELs, sequence variation within hub genes was limited, and allele-specific expression analysis revealed no significant allelic imbalance. In contrast, presence–absence variation (PAV) analysis identified structural differences affecting MFS transporter genes, which were absent in Le_S but present and upregulated in Le_L. Collectively, these findings suggest that structural genomic variation, particularly involving transporter genes, may play a more prominent role than sequence-level variation in driving phenotypic divergence. This study provides new insights into the genetic basis of strain-specific traits in *L. edodes* and highlights the importance of integrating multi-level genomic analyses.

## 1. Introduction

*Lentinula edodes* (shiitake mushroom) is one of the most widely cultivated edible fungi worldwide and represents a major agricultural commodity, particularly in East Asia [1]. In addition to its economic importance, *L. edodes* has attracted considerable scientific interest due to its nutritional value and well-documented medicinal properties, including immunomodulatory and antitumor activities associated with bioactive compounds such as lentinan [1,2]. As a model basidiomycete, it has also been extensively studied to understand the genetic and molecular mechanisms underlying fungal development and differentiation [3].

The fruiting body of *L. edodes* is a highly organized multicellular structure formed through a series of tightly regulated developmental stages, including mycelial growth, hyphal aggregation, primordium formation, and differentiation into mature structures composed of the stipe and pileus [3,4]. Among these, pileus development is particularly critical, as it directly influences spore production, reproductive success, and commercial value [4]. However, fruiting body formation is highly sensitive to both environmental and genetic factors, and abnormal morphologies, such as reduced or absent pileus structures, are frequently observed during cultivation [4]. These abnormalities not only reduce yield and market quality but also reflect underlying disruptions in developmental regulation.

Previous studies have attempted to elucidate the molecular basis of fruiting body development in *L. edodes* and other basidiomycetes using transcriptomic approaches [5,6,7,8]. These studies have identified large-scale changes in gene expression associated with developmental transitions, highlighting the involvement of genes related to cell wall remodeling, carbohydrate metabolism, signal transduction, and stress response [6,7,8,9]. In particular, differential expression analyses have revealed that genes associated with transcriptional regulation and metabolic processes play important roles during different stages of mushroom development [7,8].

In parallel, advances in genome sequencing technologies have enabled the identification of genetic variation at the sequence level, including single nucleotide polymorphisms (SNPs) and insertion–deletion mutations (INDELs) [10]. Comparative genomic studies have demonstrated that such variations can contribute to phenotypic diversity among fungal strains [6,11]. However, sequence-level variation alone does not always fully explain observed phenotypic differences, suggesting that additional layers of regulation may be involved [11].

More recently, structural genomic variation, including presence–absence variation (PAV) and copy number variation (CNV), has emerged as an important factor influencing phenotypic diversity in various organisms [12,13]. These structural variations can alter gene content and gene dosage, potentially leading to functional differences that cannot be captured by expression analysis alone [13]. Despite their potential importance, the contribution of structural variation to phenotypic differences in *L. edodes* remains largely unexplored.

In addition, network-based approaches such as protein–protein interaction (PPI) analysis and hub gene identification have been widely used to identify key regulatory genes within complex biological systems [14]. Hub genes, which occupy central positions within interaction networks, are often considered critical for maintaining functional integrity and coordinating biological processes [15]. However, the extent to which such hub genes are conserved across strains or affected by genomic variation has not been systematically investigated in *L. edodes*.

Taken together, previous studies have provided valuable insights into transcriptional and genomic variation, whereas they have largely examined these factors in isolation. A comprehensive understanding of phenotypic divergence requires the integration of multiple layers of information, including gene expression, network structure, evolutionary conservation, and genomic variation.

In this study, we investigated the molecular basis of phenotypic differences between two *L. edodes* strains, Le_L and Le_S, which exhibit distinct fruiting body morphologies, including differences in pileus development. We performed an integrative analysis combining RNA sequencing, differential expression analysis, protein interaction network construction, hub gene identification, ortholog analysis across 33 strains, genome-wide variant detection, allele-specific expression (ASE) analysis, and presence–absence variation (PAV) analysis. Specifically, we aimed to (i) characterize transcriptional and functional differences between the two strains, (ii) assess the conservation and variability in hub genes across multiple strains, and (iii) determine the relative contributions of sequence-level variation and structural genomic variation to phenotypic divergence. Through this multi-layered approach, we provide new insights into the genetic mechanisms underlying strain-specific traits in *L. edodes*.

## 2. Materials and Methods

### 2.1. Fungal Strains and RNA Sequencing

Two *L. edodes strains*, CBMLE44 (Le_L) and KMCC05708 (Le_S), exhibiting distinct fruiting body morphologies were used in this study. These strains were obtained from the Mushroom Research Division of the Rural Development Administration (RDA), Republic of Korea. The Le_L strain produces fruiting bodies with a well-developed pileus, whereas the Le_S strain exhibits an abnormal phenotype characterized by a reduced or absent pileus. These two strains were selected for comparative transcriptomic and genomic analyses to investigate genetic factors associated with pileus development. Fruiting body tissues from both strains were collected for RNA sequencing and subsequent molecular analyses.

For transcriptome analysis, fruiting body tissues were collected and immediately frozen in liquid nitrogen, followed by grinding to a fine powder. Total RNA was extracted using TRIzol reagent (Thermo Fisher Scientific Korea, Seoul, Republic of Korea) according to the manufacturer’s instructions. The extracted RNA was further purified using the RNeasy Mini Kit (Qiagen Korea, Seoul, Republic of Korea) with RNase-free DNase I (Qiagen Korea) to remove genomic DNA contamination. RNA quality and integrity were evaluated using agarose gel electrophoresis and spectrophotometric measurements. High-quality RNA samples (1 μg per sample) were used for RNA sequencing library preparation, and the libraries were sequenced on an Illumina HiSeq 2000 platform (Illumina Korea, Seoul, Republic of Korea) to generate paired-end reads. Three biological replicates were prepared for each strain.

### 2.2. Transcriptome Assembly, Quantification, and Differential Expression Analysis

Quality-filtered RNA-seq reads were trimmed to remove adapter sequences and low-quality bases using a Phred quality score threshold of 30. De novo transcriptome assembly was performed using the Trinity pipeline (version 2.15.0), which reconstructs transcript sequences through the Inchworm, Chrysalis, and Butterfly modules [16]. Putative coding regions within assembled transcripts were predicted using TransDecoder (version 5.5.0). To reduce redundancy, transcript sequences were clustered and deduplicated using CD-HIT (version 4.8.1) [17].

Transcript abundance was quantified using Salmon, which estimates transcript expression levels using a quasi-mapping strategy and probabilistic modeling approach [18]. Differential gene expression analysis between the Le_L and Le_S strains was performed using DESeq2 in the R statistical environment [19]. Gene expression counts were normalized using size factors estimated by DESeq2, and differential expression was evaluated using a negative binomial generalized linear model [19]. Genes with an adjusted *p*-value (Benjamini–Hochberg false discovery rate) < 0.05 and |log_2_ fold change| ≥ 1 were considered significantly differentially expressed genes (DEGs).

Gene expression patterns were visualized using heatmaps, volcano plots, and boxplots based on variance-stabilizing transformed (VST) expression values generated by DESeq2 [19]. Data visualization was performed using the ggplot2 and pheatmap packages in R [20,21].

### 2.3. Functional Annotation and Enrichment Analysis

Functional annotation of predicted protein sequences was performed using InterProScan, which integrates multiple protein signature databases including Pfam, SUPERFAMILY, and CDD to identify conserved protein domains and functional features [22]. Gene Ontology (GO) annotations associated with InterPro entries were retrieved from the InterProScan output [22].

KEGG Orthology (KO) assignments were performed using KofamScan, which identifies KEGG orthologs using profile hidden Markov models (HMMs) and adaptive score thresholds [23].

Functional enrichment analyses of differentially expressed genes were conducted using the clusterProfiler package implemented in R [24]. GO enrichment analysis was performed using GO annotations obtained from InterProScan results, and KEGG pathway enrichment analysis was conducted using KO assignments derived from KofamScan [22,23,24]. Statistical significance of enriched terms was evaluated using the hypergeometric test, and multiple testing correction was performed using the Benjamini–Hochberg method. Terms with an adjusted *p*-value < 0.05 were considered significantly enriched. Data processing and visualization were performed using the data.table, ggplot2, enrichplot, and GO.db packages [20,25,26,27].

### 2.4. Protein–Protein Interaction Network and Hub Gene Identification

Protein–protein interaction networks were constructed using the STRING database, which integrates experimentally validated and predicted protein associations [14]. The resulting interaction networks were visualized and analyzed using Cytoscape software (v 3.10.3) [28].

Hub genes within the interaction networks were identified using the CytoHubba plugin, which ranks nodes based on network topology parameters such as maximal clique centrality (MCC), degree, and betweenness centrality [29]. In addition, highly interconnected network modules were detected using the MCODE plugin in Cytoscape [30].

### 2.5. Variant Detection and Structural Variation Analysis

Whole-genome sequencing reads from the Le_L and Le_S strains, together with additional *L. edodes* isolates, were mapped to the Sanjo502-19 reference genome to identify genomic variants. Variant calling was performed to detect single nucleotide polymorphisms (SNPs) and insertion–deletion mutations (INDELs).

Genomic variants located within annotated genes were identified using gene annotations generated by funannotate, a fungal genome annotation pipeline [31]. Structural variation analyses were conducted to identify presence/absence variation (PAV), copy number variation (CNV), and gene deletion events relative to the Sanjo502-19 reference genome.

### 2.6. Ortholog Identification Across Multiple Strains

Orthologous gene groups across multiple *L. edodes* strains were inferred using OrthoFinder [32]. Predicted protein sequences from the Le_L and Le_S strains, together with genome data from 31 publicly available *L. edodes* isolates retrieved from the NCBI Sequence Read Archive (SRA), were used for orthology analysis. These isolates included SRR21185205, SRR21185215, SRR21185219, SRR21185260, SRR21185262, SRR21185264, SRR21185270, SRR21185272, SRR21185274, SRR21185280, SRR21185288, SRR21185292, SRR21185296, SRR21185298, SRR21185308, SRR21185310, SRR21185316, SRR21185318, SRR21185322, SRR21185324, SRR21185332, SRR21185336, SRR21185344, SRR21185356, SRR21185377, SRR21185379, SRR21185387, SRR21185402, SRR21185403, SRR23604078, and Sanjo502-19 genome assembly (GCA_030770125.1).

### 2.7. Allele-Specific Expression Analysis

Allele-specific expression (ASE) analysis was performed to evaluate the transcriptional impact of SNP variants identified within hub genes. RNA-seq reads from the Le_L and Le_S strains were aligned to the *L. edodes* Sanjo502-19 reference genome using STAR, a splice-aware RNA-seq aligner optimized for accurate transcriptome read mapping [33]. Only uniquely mapped reads were retained for downstream analysis. Mapped reads overlapping previously identified SNP loci were extracted to determine allele-specific read counts. For each SNP position, the number of reads supporting the reference and alternative alleles was calculated. Allelic expression fractions were then determined as the proportion of reads supporting the alternative allele relative to the total number of reads covering the variant site. ASE analysis was used to assess whether SNP variants within hub genes affected allele-specific transcription in the two strains.

### 2.8. Statistical Analysis

All statistical analyses were conducted in the R environment. Differential gene expression analysis was performed using DESeq2 based on a negative binomial generalized linear model. *p*-values were adjusted using the Benjamini–Hochberg method, and genes with an adjusted *p*-value < 0.05 and |log_2_ fold change| ≥ 1 were considered significantly differentially expressed. Functional enrichment analyses were conducted using hypergeometric tests implemented in the clusterProfiler package, and multiple testing correction was performed using the Benjamini–Hochberg method. Terms with adjusted *p*-values < 0.05 were considered statistically significant. For visualization, variance-stabilizing transformed (VST) expression values generated by DESeq2 were used for heatmaps and boxplots. All statistical thresholds applied in this study are described in the corresponding sections and figure legends.

### 2.9. Software and Database Availability

Publicly accessible databases and web resources used in this study, including STRING, KEGG, and InterPro-related annotation resources, were accessed between August 2025 and January 2026, and the corresponding references or URLs are provided in the References section. Software versions used in the analyses are reported in the relevant subsections whenever available.

## 3. Results

### 3.1. Morphological Differences Between the Le_L and Le_S Strains

**Figure 1:**
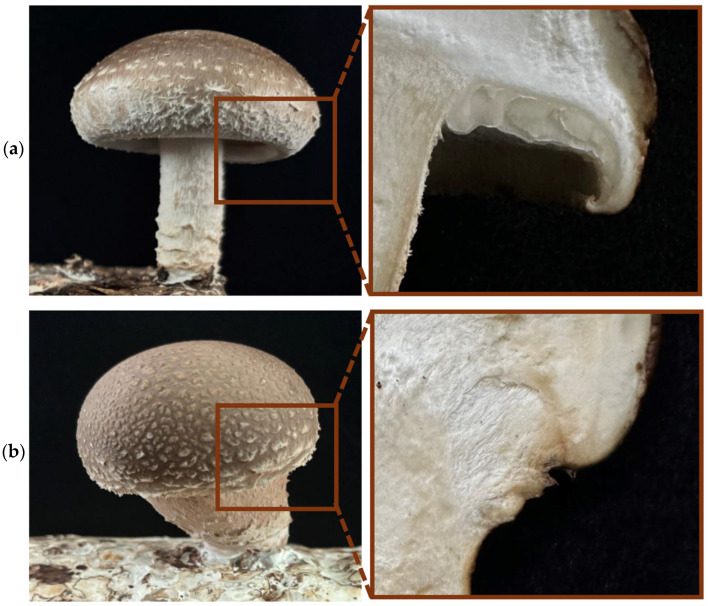
Morphological differences in fruiting bodies between the Le_L and Le_S strains of *L. edodes*. Representative images showing the fruiting body morphology of the two strains. The Le_L strain (**a**) produces normal fruiting bodies with a well-developed pileus, whereas the Le_S strain (**b**) exhibits an abnormal phenotype with a reduced or absent pileus.

Clear morphological differences were observed between the two *L. edodes* strains used in this study. The Le_L strain produced typical fruiting bodies with a well-developed pileus and normal pileus morphology, whereas the Le_S strain displayed a distinct abnormal phenotype characterized by a markedly reduced or nearly absent pileus. As shown in Figure 1, this difference was not limited to overall fruiting body appearance, but was also evident in the local structure around the pileus margin, indicating altered pileus formation rather than simple size reduction.

Because the pileus is a major reproductive structure involved in spore production and dispersal, the reduced or absent pileus phenotype of Le_S suggests disruption of developmental programs required for normal fruiting body differentiation. The stable contrast between Le_L and Le_S therefore provided an appropriate system for investigating the molecular mechanisms associated with pileus formation in *L. edodes*.

### 3.2. Transcriptome Profiling and Functional Enrichment Analysis of Differentially Expressed Genes

To identify transcriptional differences associated with the contrasting fruiting body phenotypes, RNA sequencing was performed using three biological replicates for each strain. Differential expression analysis identified a total of 8541 transcripts with significant expression differences between Le_L and Le_S (|log_2_ fold change| ≥ 1, adjusted *p*-value < 0.05; Table S1), indicating extensive transcriptional divergence between the two strains.

To functionally characterize the transcriptome, predicted proteins derived from the Trinity assembly were annotated using InterProScan and KofamScan. InterProScan assigned conserved domains to 3049 transcripts (Table S2), while KEGG Orthology (KO) annotation assigned 3424 transcripts to known functional categories (Table S3). Integration of differential expression and annotation data identified 207 functionally enriched differentially expressed transcripts, which were subsequently used for GO and KEGG enrichment analyses (Table S4).

**Figure 2:**
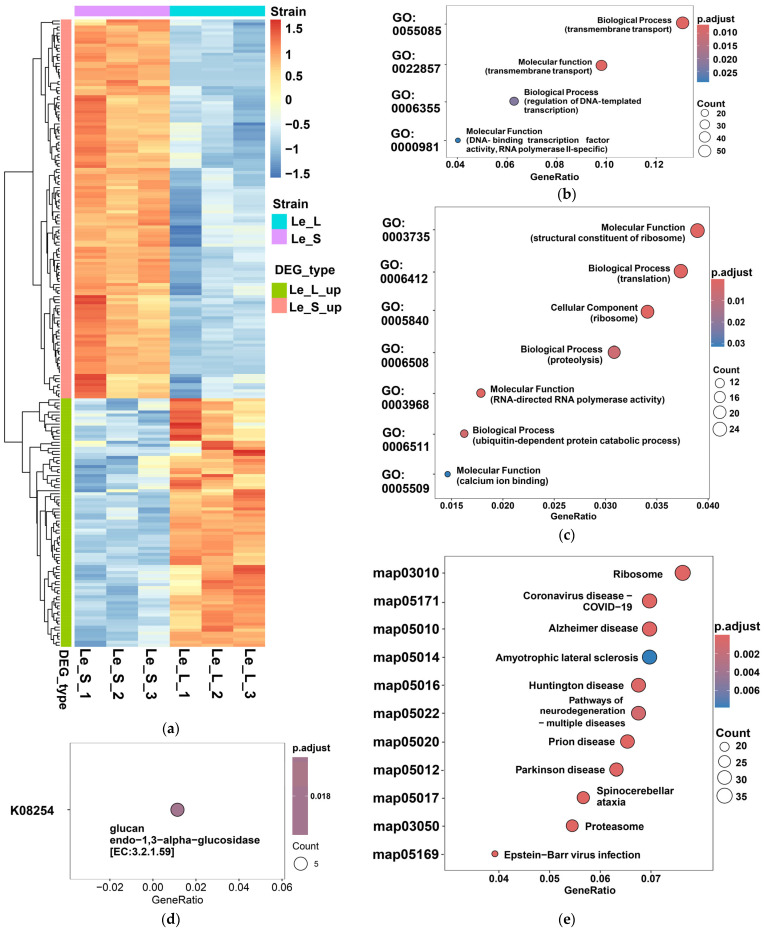
Functional enrichment analysis of differentially expressed genes between the Le_L and Le_S strains. (**a**) Heatmap showing the expression patterns of differentially expressed genes associated with enriched functional categories between the Le_L and Le_S strains. (**b**) Gene Ontology (GO) enrichment analysis of genes upregulated in Le_L. (**c**) GO enrichment analysis of genes upregulated in Le_S. (**d**) KEGG orthology enrichment analysis of Le_L-upregulated genes. (**e**) KEGG pathway enrichment analysis of Le_S-upregulated genes.

Hierarchical clustering of these enriched transcripts revealed an overall separation between Le_L and Le_S samples (Figure 2a). Although some variation was observed among biological replicates within each strain, the major expression pattern remained strain-dependent, supporting the presence of distinct transcriptional programs associated with the contrasting phenotypes. The heatmap also showed two major expression blocks, corresponding to transcripts preferentially upregulated in Le_L or in Le_S, respectively, highlighting the presence of sharply contrasting functional programs in the two strains.

GO enrichment analysis further clarified these differences. Genes upregulated in Le_L were significantly enriched in terms related to transmembrane transport, transmembrane transporter activity, and DNA-binding transcription factor activity, RNA polymerase II-specific (Figure 2b). These categories indicate that the Le_L strain is characterized by active transport processes and regulatory functions, consistent with a physiologically dynamic developmental state requiring metabolite exchange and transcriptional coordination during normal pileus formation.

In contrast, genes upregulated in Le_S were strongly enriched in GO terms associated with structural constituent of ribosome, translation, and ribosome (Figure 2c). Additional enriched categories included proteolysis and ubiquitin-dependent protein catabolic process, suggesting enhanced protein turnover in addition to elevated translational activity. This enrichment profile indicates that Le_S is characterized by a transcriptomic state dominated by ribosome-related and protein metabolism-associated processes [9], which differs markedly from the broader transport- and regulation-related program observed in Le_L.

KEGG-based analyses were consistent with the GO results. In Le_L, KEGG orthology enrichment highlighted genes associated with glucan metabolism, including glucan endo-1,3-alpha-glucosidase (Figure 2d), suggesting activation of cell wall remodeling and carbohydrate metabolism. In Le_S, KEGG pathway enrichment was dominated by ribosome-associated functions, with additional signals related to protein turnover and proteostasis (Figure 2e). Although several enriched KEGG pathway labels are represented as disease-associated categories, these primarily reflect conserved core biological processes such as translation, protein folding, and degradation. Therefore, the enrichment pattern was interpreted in terms of these underlying functional processes rather than their nominal pathway names. The repeated recovery of ribosome-associated functions in both GO and KEGG analyses supports the conclusion that translational machinery is a dominant feature of the Le_S transcriptional program.

Taken as a whole, these transcriptomic data indicate that the Le_L strain is associated with a developmentally broader expression program involving transport, metabolism, and transcriptional regulation, whereas the Le_S strain exhibits a more restricted but strongly amplified program centered on ribosome-related processes. This contrast suggests that abnormal pileus formation in Le_S is accompanied by a major shift in transcriptional priority, from transport-related and regulatory functions toward protein synthesis-related activity.

### 3.3. Protein Interaction Network Analysis and Identification of Hub Genes

**Figure 3:**
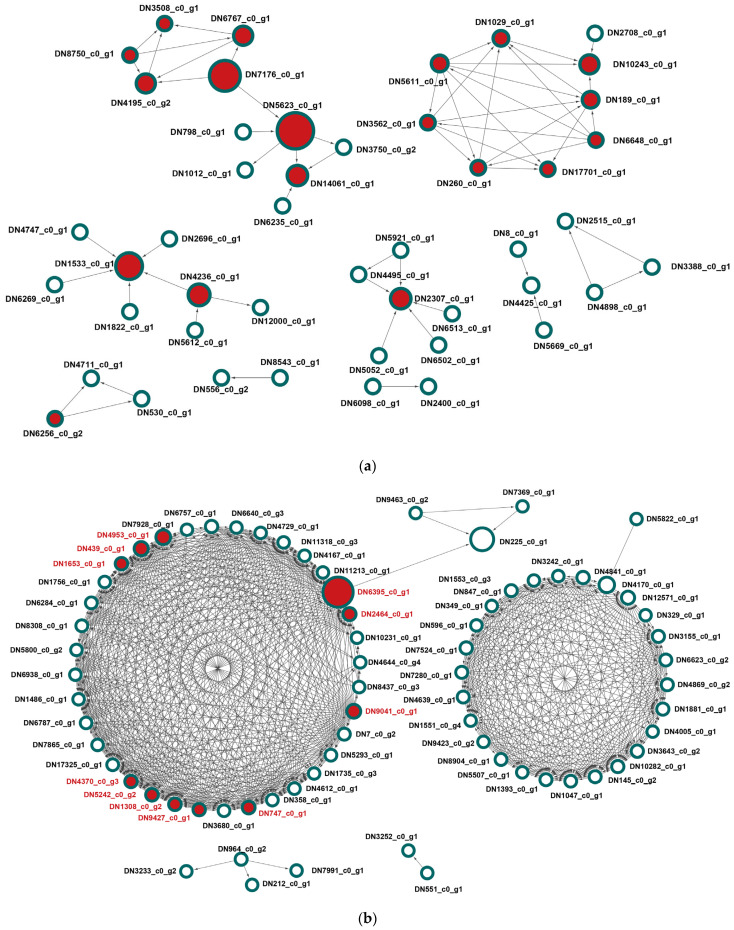
STRING-based protein–protein interaction networks of differentially expressed genes in the Le_L and Le_S strains. (**a**) Interaction network constructed from genes upregulated in Le_L. (**b**) Interaction network constructed from genes upregulated in Le_S. Nodes represent proteins and edges indicate predicted protein–protein interactions derived from the STRING database. Hub genes identified by CytoHubba analysis are highlighted in red. Node size is proportional to the degree value calculated by CytoHubba.

To further identify genes that may play central roles within the strain-specific transcriptional programs, protein–protein interaction (PPI) networks were constructed separately for the Le_L-upregulated and Le_S-upregulated DEG sets using STRING. The resulting networks revealed clear differences in both topology and functional composition between the two strains (Figure 3a,b).

The network derived from Le_L-upregulated genes showed several interconnected but relatively modular subnetworks (Figure 3a). Many of the central nodes in this network corresponded to major facilitator superfamily (MFS) transporters, sugar transporters, Zn(2)-Cys(6) transcription factors, and genes associated with carbohydrate metabolism. The presence of these transporter- and regulator-related nodes near the center of the network suggests that transport capacity, metabolic flexibility, and transcriptional control are key features of the Le_L developmental program.

By contrast, the network generated from Le_S-upregulated genes displayed a markedly denser and more highly interconnected structure (Figure 3b). This network was dominated by genes encoding ribosomal proteins and translation-associated components, which formed a tightly connected interaction module. Such dense connectivity is typical of genes functioning within the same macromolecular complex [15] and indicates strong co-regulation of the ribosomal machinery in Le_S.

To identify the most influential nodes within these networks, hub gene analysis was performed using CytoHubba, and densely connected modules were evaluated using MCODE. The resulting hub genes are summarized in Table 1, which lists transcript identifiers, expression changes, and topological parameters including MCC, degree, betweenness, and MCODE cluster assignment.

In the Le_S-upregulated network, hub genes were overwhelmingly composed of ribosomal proteins, including DN1308_c0_g2 (large subunit ribosomal protein L28e), DN1653_c0_g1 (large subunit ribosomal protein LP1), DN2464_c0_g1 (ribosomal protein S5, N-terminal domain), DN439_c0_g1 (ribosomal protein L23), DN4953_c0_g1 (ribosomal protein L14p/L23e), DN5242_c0_g2 (ribosomal protein L14), DN9041_c0_g1 (large subunit ribosomal protein L10Ae), and DN9427_c0_g1 (ribosomal protein L6) (Table 1). These genes exhibited both high centrality and strong upregulation in Le_S, indicating that ribosome-related processes are not only enriched at the pathway level but are also central to the overall regulatory network.

In contrast, hub genes in the Le_L-upregulated network were more functionally diverse. They included multiple MFS transporters such as DN1029_c0_g1, DN17701_c0_g1, DN189_c0_g1, DN260_c0_g1, DN3562_c0_g1, DN5611_c0_g1, DN6648_c0_g1, DN6767_c0_g1, DN7176_c0_g1, and DN8750_c0_g1, together with DN14061_c0_g1 and DN5623_c0_g1 annotated as sugar transporters, DN1533_c0_g1 and DN6256_c0_g2 annotated as Zn(2)-Cys(6) transcription factors, and DN4236_c0_g1 annotated as glycosyl hydrolase family 71 (Table 1). This pattern indicates that the Le_L network is organized around transport and regulatory functions rather than a single dominant biosynthetic module.

These network results are important because they refine the enrichment analysis by highlighting not only which functional categories are overrepresented but also which genes occupy central regulatory positions within those categories. In particular, the strong dominance of ribosomal hub genes in Le_S suggests a coordinated and possibly compensatory upregulation of translational machinery, whereas the predominance of transporter- and regulator-related hub genes in Le_L is more consistent with active developmental coordination during normal pileus formation.

### 3.4. Expression Patterns of Hub Genes Identified from Network Analysis

To validate the strain-specific expression patterns of the hub genes identified above, their expression profiles were examined using heatmap, volcano plot, and boxplot visualizations.

**Figure 4:**
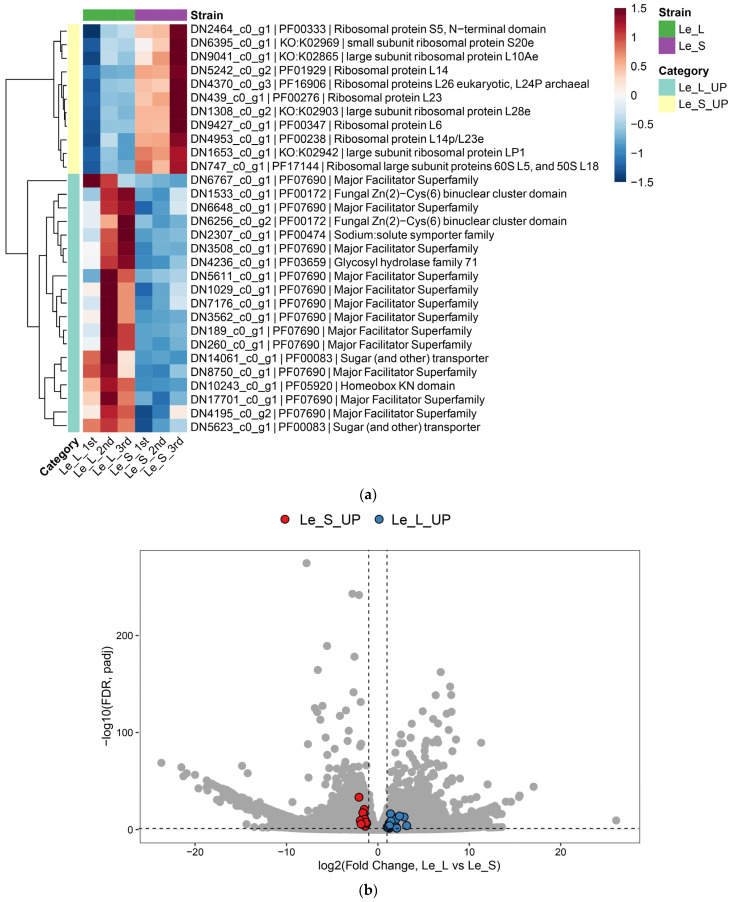
Differential expression patterns of hub genes between the Le_L and Le_S strains. (**a**) Heatmap showing the expression patterns of hub genes across biological replicates of Le_L and Le_S. (**b**) Volcano plot highlighting hub genes among differentially expressed transcripts.

The heatmap of hub genes showed clear group-level separation between Le_L and Le_S samples (Figure 4a). Most Le_S-upregulated hub genes exhibited higher expression in Le_S, whereas most Le_L-upregulated hub genes showed higher expression in Le_L. Although replicate-level variation was present, the overall group-specific expression patterns were maintained.

The volcano plot further illustrated that these hub genes were not marginally differentially expressed but instead represented a subset of transcripts with both strong fold changes and high statistical significance (Figure 4b). Le_S-associated hub genes were concentrated on the negative side of the log_2_ fold change axis, reflecting their higher expression in Le_S, whereas Le_L-associated hub genes were concentrated on the positive side, consistent with their higher expression in Le_L.

**Figure 5:**
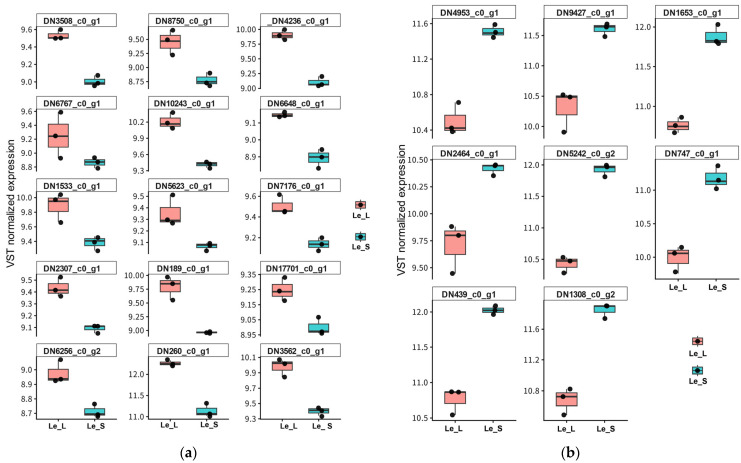
Boxplot visualization of VST-normalized expression levels of hub genes between the Le_L and Le_S strains. (**a**) Expression levels of hub genes upregulated in Le_L (Le_L_UP) across biological replicates of Le_L and Le_S. (**b**) Expression levels of hub genes upregulated in Le_S (Le_S_UP) across biological replicates of Le_L and Le_S. Expression values represent variance-stabilizing transformation (VST)-normalized RNA-seq expression levels.

These patterns were further supported by boxplot analysis of VST-normalized expression values (Figure 5a,b). Le_L-upregulated hub genes such as DN8750_c0_g1, DN4236_c0_g1, DN6767_c0_g1, DN1533_c0_g1, DN5623_c0_g1, DN189_c0_g1, and DN260_c0_g1 showed consistently higher expression in Le_L than in Le_S (Figure 5a). In contrast, Le_S-upregulated hub genes such as DN4953_c0_g1, DN9427_c0_g1, DN1653_c0_g1, DN2464_c0_g1, DN5242_c0_g2, DN747_c0_g1, DN439_c0_g1, and DN1308_c0_g2 showed consistently higher expression in Le_S (Figure 5b).

Despite some replicate-level variation, the boxplot analyses supported the overall distinction between the transporter/regulator-centered program in Le_L and the ribosome-centered program in Le_S. The agreement among heatmap, volcano plot, and boxplot analyses strengthens the conclusion that these hub genes are biologically meaningful candidates associated with the contrasting phenotypes.

### 3.5. Conservation and Expression Patterns of Hub Genes Across L. edodes Strains

To determine whether the hub genes identified from transcriptome and network analyses are conserved across *L. edodes* strains, ortholog analysis was performed using OrthoFinder across 33 genomes. The overall orthogroup assignments are summarized in Table S5, providing a genome-wide overview of gene conservation.

Detailed examination of hub genes revealed that the majority were assigned to orthogroups classified as “Shared”, indicating that these genes are widely distributed across the analyzed strains (Table S6). Most hub genes were detected in nearly all genomes, supporting their classification as core components of the *L. edodes* gene repertoire [8]. This pattern was consistently observed for both phenotype-associated groups, including transporter- and regulatory-related genes in the Le_L_UP group and ribosomal protein genes in the Le_S_UP group.

In several orthogroups, the total number of gene copies exceeded the number of analyzed strains (e.g., copy_range up to 34 despite 33 genomes). This pattern reflects gene duplication events in specific strains rather than the inclusion of additional genomes, indicating that certain hub genes have undergone limited copy number expansion. However, no meaningful differences in gene copy number were observed between the two focal strains (Le_L and Le_S), as indicated by identical copy values (copy_L = 0 and copy_S = 0 across all hub genes). These values reflect the absence of detectable copy number variation within each orthogroup, rather than the absence of the genes themselves, suggesting that large-scale copy number variation does not underlie the phenotypic divergence between the two strains.

Although most hub genes showed widespread conservation, a subset exhibited reduced presence frequencies across strains. For example, DN3508_c0_g1 was detected in only a limited number of genomes, while DN10243_c0_g1 and DN6256_c0_g2 showed intermediate conservation levels. These patterns suggest that while the majority of hub genes represent conserved functional components, some may be subject to lineage-specific variation or functional diversification.

Overall, the ortholog analysis indicates that the core hub gene set is structurally conserved across *L. edodes* strains. Therefore, the phenotypic differences observed between Le_L and Le_S are unlikely to be explained by gene presence–absence or copy number variation in most hub genes. Instead, these results support a model in which phenotypic divergence is primarily driven by differences in gene regulation and network-level expression dynamics, rather than by large-scale changes in gene content.

### 3.6. Genome-Wide Variant Analysis and Identification of Variants in Hub Genes

To investigate sequence-level genomic differences between the strains, whole-genome variant analysis was performed by aligning reads from Le_L, Le_S, and 30 additional isolates to the Sanjo502-19 reference genome. This analysis identified 7931 SNPs (Table S7) and 1149 INDELs (Table S8), indicating substantial genome-wide variation within the analyzed *L. edodes* population.

To assess the potential functional relevance of these variants in the Le_S strain, SNPs and INDELs were mapped onto gene features predicted by funannotate. A total of 609 genes contained SNPs (Table S9) and 126 genes contained INDELs (Table S10) within gene-associated regions, including exons, introns, and untranslated regions. This indicates that a considerable number of genes in Le_S harbor sequence-level variation.

However, when the analysis was restricted to hub genes, only a very limited number of variants were identified. As summarized in Table S11, only four hub genes contained variants within gene features, indicating that the genes most central to the expression network remain largely conserved despite extensive genome-wide variation.

Detailed inspection of these hub-associated variants showed that they were located in genes related to transcriptional regulation, transport, and ribosomal function (Table 2). Specifically, variants were identified in FUN_005650 and FUN_010198 annotated as Zn(2)-Cys(6) transcription factor-related genes, FUN_005831 annotated as a sugar transporter, and FUN_005882 annotated as a ribosomal protein S5-related gene. The variant types included intronic substitutions and synonymous exon substitutions, suggesting limited potential for direct protein-disrupting effects.

Importantly, no frameshift-causing INDELs, nonsense variants, or other clearly disruptive mutations were detected within these hub genes. This suggests that, although sequence variation exists, it is unlikely to represent a major mechanism of structural inactivation in the central regulatory genes identified here. Instead, the pattern is more consistent with modest sequence-level diversification superimposed on an otherwise conserved hub gene set.

### 3.7. Allele-Specific Expression Analysis of Variants in Hub Genes

To determine whether the SNPs identified within hub genes affected transcription in an allele-specific manner, ASE analysis was performed using RNA-seq reads mapped to the Sanjo502-19 reference genome. The results are summarized in Table 3.

At the examined hub gene loci, the Le_L strain consistently exhibited homozygous reference alleles (0/0) with no detectable alternative allele reads, indicating the absence of allelic heterogeneity at these positions. In contrast, the Le_S strain showed heterozygous genotypes (0/1) at several SNP positions, particularly in FUN_005650, FUN_005831, and FUN_005882.

Despite this heterozygosity, the alternative allele fractions in Le_S were generally close to 0.5, as shown in Table 3, indicating balanced expression of both alleles. For example, FUN_005650 showed alternative allele fractions of approximately 0.41–0.48 across replicates, while FUN_005882 showed values of approximately 0.52–0.53. Although one locus in FUN_005831 showed a somewhat elevated value in a low-depth sample, no consistent allelic bias was observed across replicates.

These data indicate that the SNPs present within the analyzed hub genes do not produce strong monoallelic or biased expression patterns. Therefore, the observed transcriptional differences between Le_L and Le_S are unlikely to be driven primarily by cis-acting allele-specific effects at these SNP loci. Instead, broader regulatory differences between the strains appear to be more important in shaping hub gene expression.

### 3.8. Presence–Absence Variation Analysis of Hub Genes

To evaluate whether structural genomic differences contribute to the phenotypic divergence between Le_L and Le_S, presence–absence variation (PAV) analysis was performed across *L. edodes* strains. Most hub genes identified from transcriptome and network analyses were structurally conserved across strains, consistent with the orthogroup analysis. However, a limited subset of genes exhibited PAV or reduced copy number in specific strains.

Notably, the clearest PAV signal involved genes associated with membrane transport, particularly members of the Major Facilitator Superfamily (MFS). As shown in Table 4, FUN_003231 annotated as an MFS general substrate transporter and FUN_003565 annotated as a Major Facilitator Superfamily protein were absent in Le_S but present in Le_L and in the other analyzed strains. Because these genes were also validated by ortholog analysis across multiple genomes, their absence in Le_S is unlikely to reflect an annotation artifact and instead indicates genuine strain-specific structural variation.

This finding is important because MFS-related genes emerged repeatedly throughout the study. They were enriched among Le_L-upregulated hub genes, occupied central positions in the Le_L interaction network (Figure 3a and Table 1), showed consistently higher expression in Le_L (Figure 4a and Figure 5a), and, in a subset of cases, were structurally absent in Le_S (Table 4). Thus, among the functional gene classes identified here, MFS transporters are the only category supported by both transcriptional and structural evidence.

Although the number of PAV-affected hub genes was limited, this pattern suggests that altered transporter gene content may contribute to the abnormal pileus phenotype in Le_S. Given the established roles of membrane transporters in nutrient uptake [4], metabolite distribution, and developmental homeostasis, the loss of specific MFS-related genes may affect the physiological conditions required for normal pileus development. At the same time, because most hub genes remained conserved and no direct functional validation was performed, these PAV results should be interpreted as strong candidate evidence rather than definitive proof of causality.

Overall, the PAV analysis indicates that structural variation is not widespread across the hub gene set but selectively affects a small number of transporter-related genes. This makes MFS-associated structural variation one of the most compelling candidates for contributing to phenotypic divergence between Le_L and Le_S.

## 4. Discussion

In this study, we performed an integrative analysis combining transcriptome profiling, network-based hub gene identification, comparative ortholog analysis across multiple strains, and genome-wide variant detection to elucidate the molecular basis underlying phenotypic differences between the Le_L and Le_S strains of *L. edodes*.

A prominent feature of the transcriptomic differences was the contrasting functional profiles between the two strains. Genes upregulated in Le_S were predominantly associated with ribosomal proteins and translational machinery, indicating enhanced protein synthesis capacity. In contrast, Le_L-upregulated genes were enriched in transporters, transcription factors, and metabolic enzymes, reflecting broader functional activities related to nutrient transport and regulatory control. These differences suggest that the two strains occupy distinct physiological states and are consistent with previous transcriptomic studies showing that fungal development involves dynamic shifts between translational activity and regulatory or metabolic programs [9,34,35].

A biologically plausible interpretation of our findings is that transporter-related genes, particularly members of the major facilitator superfamily (MFS) and sugar transporter families, may play a critical role in pileus development by regulating the intracellular and intercellular distribution of nutrients and metabolites. Fruiting body development requires coordinated cellular differentiation, metabolic remodeling, and environmental responsiveness. Disruption of transporter activity could therefore alter physiological balance, nutrient allocation, and osmotic regulation, ultimately impairing normal pileus formation. The repeated identification of transporter-related genes across differential expression, network centrality, and structural variation analyses strongly supports their potential role as key contributors to the pileus-deficient phenotype.

Network analysis further highlighted clear differences in the organization of strain-specific transcriptional programs. Hub genes in Le_S were largely composed of ribosomal proteins forming a highly interconnected module, indicating strong coordination of translation-related processes. Such network structures are characteristic of genes functioning within tightly regulated macromolecular complexes and are often associated with essential cellular functions [15,36,37]. In contrast, hub genes in Le_L were functionally diverse and included multiple Major Facilitator Superfamily (MFS) transporters as well as transcriptional regulators. This contrast suggests that Le_S prioritizes translational capacity, whereas Le_L relies on more distributed regulatory and transport mechanisms.

Ortholog analysis across 33 *L. edodes* strains showed that most hub genes are widely conserved, indicating that they represent core components of the species gene repertoire. This pattern is consistent with previous genome-wide analyses of *L. edodes*, which revealed overall conservation of gene content alongside localized genomic variation [8,38]. Despite this structural conservation, variation in gene expression and copy number distribution was observed among strains, suggesting that phenotypic divergence is more strongly influenced by regulatory differences than by gene presence or absence. This interpretation aligns with previous studies demonstrating that regulatory variation can play a dominant role in shaping phenotypic diversity [11].

Genome-wide variant analysis identified substantial numbers of SNPs and INDELs across strains, whereas only a small subset of hub genes contained coding-region variants. In addition, allele-specific expression analysis did not reveal consistent allelic imbalance at these loci. These findings indicate that sequence-level variation alone is unlikely to account for the observed transcriptional differences and instead point to higher-order regulatory mechanisms as primary drivers of expression divergence [11].

In contrast, presence–absence variation analysis revealed structural differences affecting a limited number of genes, particularly those encoding MFS transporters. The absence of specific MFS genes in the Le_S strain, combined with their higher expression in Le_L, suggests that structural variation in transporter genes may contribute to phenotypic divergence. MFS transporters are known to play critical roles in substrate transport, environmental adaptation, and metabolic regulation in fungi [4,39,40], supporting their potential involvement in fruiting body development.

Taken together, these results indicate that phenotypic differences between the two *L. edodes* strains are not primarily driven by large-scale differences in gene content or coding sequence variation. Instead, they are more likely to arise from coordinated changes in gene regulation and network organization, with structural variation in transporter genes representing a key contributing factor. Further functional studies will be required to validate the causal roles of these candidate genes and to clarify the regulatory mechanisms underlying strain-specific developmental traits. We acknowledge that this study is primarily based on integrative bioinformatic analyses and does not include direct experimental validation. Therefore, the identified hub genes and transporter-associated loci should be considered strong candidate factors rather than definitive causal determinants. Future studies involving experimental validation, such as qPCR analysis or functional assays, will be necessary to confirm their specific roles in pileus morphogenesis.

## 5. Conclusions

In conclusion, this study provides an integrative analysis of transcriptional regulation, network structure, and genomic variation underlying phenotypic divergence in *L*. *edodes*. Our results suggest that differences in regulatory and transporter-associated gene networks, rather than sequence-level variation alone, play a central role in determining pileus morphology.

Importantly, the transporter-related hub genes and structurally variable loci identified in this study represent promising candidates for future functional validation. From an applied perspective, these findings provide a potential framework for improving strain selection, breeding strategies, and cultivation stability in *L. edodes*. The integration of transcriptomic, network, and structural variation analyses may also serve as a useful approach for identifying key determinants of developmental traits in other mushroom species.

## Figures and Tables

**Table 1:** Hub genes identified from differentially expressed transcripts based on STRING network analysis and Cytoscape CytoHubba and MCODE analyses.

DEG	Transcript ID	log_2_FC	MCC	Degree	Betweenness	MCODE Cluster	Description
Le_S _UP	DN1308_c0_g2	−1.56	9.22 × 10^13^	64	14.01	1	large subunit ribosomal protein L28e
	DN1653_c0_g1	−1.49	9.22 × 10^13^	60	12.59	1	large subunit ribosomal protein LP1
	DN2464_c0_g1	−1.37	9.22 × 10^13^	64	19.83	1	Ribosomal protein S5, N-terminal domain
	DN4370_c0_g3	−1.67	9.22 × 10^13^	64	11.00	1	Ribosomal proteins L26 eukaryotic, L24P archaeal
	DN439_c0_g1	−1.65	9.22 × 10^13^	68	32.34	1	Ribosomal protein L23
	DN4953_c0_g1	−1.42	9.22 × 10^13^	68	40.35	1	Ribosomal protein L14p/L23e
	DN5242_c0_g2	−2.07	9.22 × 10^13^	64	14.01	1	Ribosomal protein L14
	DN6395_c0_g1	−1.21	9.22 × 10^13^	68	236.58	1	small subunit ribosomal protein S20e
	DN747_c0_g1	−1.92	9.22 × 10^13^	64	16.45	1	Ribosomal large subunit proteins 60S L5, and 50S L18
	DN9041_c0_g1	−1.27	9.22 × 10^13^	68	29.74	1	large subunit ribosomal protein L10Ae
	DN9427_c0_g1	−1.84	9.22 × 10^13^	64	11.00	1	Ribosomal protein L6
Le_L _UP	DN10243_c0_g1	1.85	7	8	14		Homeobox KN domain
	DN1029_c0_g1	1.23	126	12	4	1	Major Facilitator Superfamily
	DN14061_c0_g1	1.90	3	6	18	4	Sugar (and other) transporter
	DN1533_c0_g1	1.32	5	10	36		Fungal Zn(2)-Cys(6) binuclear cluster domain
	DN17701_c0_g1	1.08	24	8	0	4	Major Facilitator Superfamily
	DN189_c0_g1	2.87	150	14	7	4	Major Facilitator Superfamily
	DN2307_c0_g1	1.28	5	10	18	6	Sodium:solute symporter family
	DN260_c0_g1	1.38	144	12	1	1	Major Facilitator Superfamily
	DN3508_c0_g1	1.96	6	6	0	2	Major Facilitator Superfamily
	DN3562_c0_g1	1.44	144	12	1	1	Major Facilitator Superfamily
	DN4195_c0_g2	1.01	8	8	14	2	Major Facilitator Superfamily
	DN4236_c0_g1	2.36	3	6	22		Glycosyl hydrolase family 71
	DN5611_c0_g1	1.26	150	14	7	1	Major Facilitator Superfamily
	DN5623_c0_g1	1.14	5	10	64	4	Sugar (and other) transporter
	DN6256_c0_g2	1.99	2	4	0	3	Fungal Zn(2)-Cys(6) binuclear cluster domain
	DN6648_c0_g1	1.28	120	10	0	1	Major Facilitator Superfamily
	DN6767_c0_g1	2.07	8	8	14	2	Major Facilitator Superfamily
	DN7176_c0_g1	1.26	3	6	48		Major Facilitator Superfamily
	DN8750_c0_g1	3.15	6	6	0	2	Major Facilitator Superfamily

**Table 2:** SNP variants identified within hub genes of the Le_S genome based on mapping to the Sanjo502-19 reference genome.

CHROM	POS	REF	ALT	Le_S Gene_ID	Le_S	Le_L	Others Strain	Annotation	DEG (Hub Gene)	Variation
ch_9	1225767	G	A	FUN_005650	G/A	G/G	G(SRR21185377;A)	Fungal Zn(2)-Cys(6) binuclear cluster domain	Le_L_UP	Exon (synonymous)
ch_7	2193207	C	T	FUN_005831	C/T	C/C	C(SRR21185336; T)	Sugar (and other) transporter	Le_L_UP	intron
ch_7	3006689	T	C	FUN_005882	T/C	T/T	T(SRR21185324; C)	Ribosomal protein S5, N-terminal domain	Le_S_UP	Exon (synonymous)
ch_7	3006825	T	C	FUN_005882	T/C	T/T	T(SRR21185324; C)		Le_S_UP	
ch_1	5218994	G	A	FUN_010198	G/A	G/G	G(SRR21185205; A)	Fungal Zn(2)-Cys(6) binuclear cluster domain	Le_L_UP	intron

**Table 3:** Allele-specific expression (ASE) analysis of SNP variants located in hub genes of the Le_S genome.

Sample	Group	CHROM	POS	Le_S Gene_ID	REF	ALT	GT	DP	REFc	ALTc	ALTfrac
Le_L-1	L	ch_9	1225767	FUN_005650	G	.	0/0	9	9		0
Le_L-1	L	ch_7	2193207	FUN_005831	C	.	0/0	4	4		0
Le_L-1	L	ch_7	3006689	FUN_005882	T	.	0/0	32	32		0
Le_L-1	L	ch_7	3006825	FUN_005882	T	.	0/0	32	32		0
Le_L-1	L	ch_1	5218994	FUN_010198	G	.	0/0	1	1		0
Le_L-2	L	ch_9	1225767	FUN_005650	G	.	0/0	23	23		0
Le_L-2	L	ch_7	2193207	FUN_005831	C	.	0/0	3	3		0
Le_L-2	L	ch_7	3006689	FUN_005882	T	.	0/0	104	104		0
Le_L-2	L	ch_7	3006825	FUN_005882	T	.	0/0	104	104		0
Le_L-2	L	ch_1	5218994	FUN_010198	G	.	./.	0	0		NA
Le_L-3	L	ch_9	1225767	FUN_005650	G	.	0/0	33	33		0
Le_L-3	L	ch_7	2193207	FUN_005831	C	.	0/0	6	6		0
Le_L-3	L	ch_7	3006689	FUN_005882	T	.	0/0	92	91		0
Le_L-3	L	ch_7	3006825	FUN_005882	T	.	0/0	92	91		0
Le_L-3	L	ch_1	5218994	FUN_010198	G	.	0/0	2	2		0
Le_S-1	S	ch_9	1225767	FUN_005650	G	A	0/1	61	32	29	0.48
Le_S-1	S	ch_7	2193207	FUN_005831	C	T	0/1	6	2	4	0.67
Le_S-1	S	ch_7	3006689	FUN_005882	T	C	0/1	199	94	105	0.53
Le_S-1	S	ch_7	3006825	FUN_005882	T	C	0/1	199	94	105	0.53
Le_S-1	S	ch_1	5218994	FUN_010198	G	.	0/0	1	1		0.00
Le_S-2	S	ch_9	1225767	FUN_005650	G	A	0/1	49	29	20	0.41
Le_S-2	S	ch_7	2193207	FUN_005831	C	.	0/0	1	0		NA
Le_S-2	S	ch_7	3006689	FUN_005882	T	C	0/1	189	91	98	0.52
Le_S-2	S	ch_7	3006825	FUN_005882	T	C	0/1	189	91	98	0.52
Le_S-2	S	ch_1	5218994	FUN_010198	G	.	./.	0	0		NA
Le_S-3	S	ch_9	1225767	FUN_005650	G	A	0/1	74	42	32	0.43
Le_S-3	S	ch_7	2193207	FUN_005831	C	.	0/0	3	2		0.00
Le_S-3	S	ch_7	3006689	FUN_005882	T	C	0/1	201	97	104	0.52
Le_S-3	S	ch_7	3006825	FUN_005882	T	C	0/1	201	97	104	0.52
Le_S-3	S	ch_1	5218994	FUN_010198	G	.	./.	0	0		NA

**Table 4:** Hub genes absent in the Le_S genome identified by PAV/CNV/deletion analysis relative to the Sanjo502-19 reference genome and validated by ortholog analysis across 30 *L. edodes* strains.

Gene_ID (Sanjo)	Orthogroup	No. of Gene		Other Strains	Annotation		
		Le_S	Le_L		DB	Accession	Description
FUN_003231	OG0005310	0	1	present in all	SUPERFAMILY	SSF103473	MFS general substrate transporter
FUN_003565	OG0005419	0	1	present in all	Pfam	PF07690	Major Facilitator Superfamily

## Data Availability

The RNA sequencing and genome sequencing datasets generated for the Le_L and Le_S strains in this study have been deposited in the NCBI Sequence Read Archive (SRA) under accession numbers SRR37738952, SRR37738953, SRR37794314–SRR37794319.

## Supplementary Materials

The following supporting information can be downloaded at: https://www.mdpi.com/article/10.3390/jof12050328/s1, Table S1: Differentially expressed transcripts between Le_L and Le_S strains identified from RNA-seq analysis (|log_2_FC| ≥ 1, adjusted *p*-value < 0.05); Table S2: InterProScan functional annotation of Trinity-derived proteins identified in the transcriptome analysis (E-value < 0.001); Table S3: KEGG Orthology (KO) annotations of Trinity transcripts identified using KofamScan based on threshold score criteria; Table S4: Functional annotation of differentially expressed transcripts used for GO and KEGG enrichment analyses between the Le_L and Le_S strains; Table S5: Orthogroup assignment of predicted genes identified by OrthoFinder analysis across *L. edodes* strains; Table S6: Orthogroup assignment and conservation patterns of hub genes across 33 *L. edodes* strains based on OrthoFinder analysis, including copy number variation and expression changes between Le_L and Le_S strains; Table S7: Genome-wide SNP variants detected in Le_L, Le_S, and 30 additional *L*. *edodes* isolates based on alignment to the *L. edodes* Sanjo502-19 reference genome; Table S8: Genome-wide INDEL variants detected in Le_L, Le_S, and 30 additional *L*. *edodes* isolates based on alignment to the *L. edodes* Sanjo502-19 reference genome; Table S9: Genomic positions of SNPs identified in the Le_S strain within gene features of Le_S genes predicted by funannotate based on the *L. edodes* Sanjo502-19 reference genome; Table S10: Genomic positions of INDELs identified in the Le_S strain within gene features of Le_S genes predicted by funannotate based on the *L. edodes* Sanjo502-19 reference genome; Table S11: Distribution of SNP and INDEL variants across Le_S genes and identification of variants occurring in hub genes.
